# Unplanned adaptations before breaking the blind

**DOI:** 10.1002/sim.5361

**Published:** 2012-06-27

**Authors:** Martin Poscha, Michael A. Proschanb

**Affiliations:** a European Medicines Agency7 Westferry Circus, E14 4HB London, U.K; b National Institute of Allergy and Infectious Diseases6700A Rockledge Drive, Bethesda, MD, U.S.A.

**Keywords:** adaptive methods, permutation tests, type I error rate control, multiple comparisons, blinded interim analysis

## Abstract

Occasionally, things go so wrong in a clinical trial that a change must be made. For example, the originally planned primary outcome may be measured completely unreliably. Is there any recourse? One may still be able to salvage the trial using a permutation test if a change is made before breaking the treatment blind. The solution is not a panacea; we discuss the limitations and legitimate grounds for criticism. Still, when it is needed, the procedure is preferable to rigid adherence to a design that makes no sense. Published 2012. This article is a US Government work and is in the public domain in the USA.

## 1 Introduction

Clinical trials sometimes undergo unplanned changes in aspects such as the population, primary end point, or analysis plan. One reason for modifying the population is to increase lagging recruitment. For instance, the Antihypertensive and Lipid Lowering Treatment to Prevent Heart Attack Trial originally did not include smoking as a qualifying risk factor but subsequently included it to boost recruitment 1. Another reason for changing the population is that interim results may definitively answer the trial question in a subgroup. A trial of lung volume reduction surgery in patients with severe emphysema determined at an interim analysis that the surgery resulted in excess mortality in patients with low forced expiratory volume (FEV _1_), so they discontinued recruitment of this subgroup 2,3. These and other examples illustrate that the population may be changed deliberately over the course of a trial.

Another modification of trial design is a change in the primary end point. The primary end point for the Raloxifene Use for the Heart trial was originally nonfatal heart attack or coronary death but was expanded to include acute coronary syndromes to increase a lower than expected event rate 4. A clinical trial using imaging techniques such as ultrasound or angiography might find that one imaging outcome is measured more reliably than another. Changing the primary end point seems drastic, but in some cases there is substantial pretrial uncertainty about which of two potential outcomes should be primary. For instance, the Women's Angiographic Vitamin and Estrogen (WAVE) trial investigators were torn between using change in minimum lumen diameter or percent stenosis to assess blockage of segments of coronary arteries 5. The Dietary Approaches to Stop Hypertension (DASH) trial investigators debated whether to assess the effect of different dietary patterns on systolic or diastolic blood pressure change as the primary outcome 6. Neither WAVE nor DASH changed primary end points, but these trials illustrate that there can be substantial uncertainty about which end point is best.

Changes in the analysis plan can also occur. The Late-Onset Treatment Study for Pompe disease, a very rare neuromuscular disorder, changed their primary analysis from a mixed model to analysis of covariance after discovering that the assumptions underlying the mixed model were violated 7. Similarly, one might want to change a parametric to nonparametric analysis after detecting outliers. Despite the best intentions of clinical trial planners, unplanned changes occur.

Unplanned changes fall outside common regulatory guidelines about adaptive methods. For example, the Food and Drug Administration and the European Medicines Agency guidelines stress that adaptive methods must be pre-planned. At the same time, these guidance documents recognize that changes made before breaking the treatment blind are much less objectionable than changes after breaking the blind. The present article considers this murky area of an *unplanned* change *before unblinding*.

The key to a valid and sensible analysis when an unplanned change is made is to find a method that controls the *conditional* type I error rate for each possible change (including no change) rather than the *unconditional* type I error rate (i.e., the error rate averaged over all possible adaptations) at the pre-specified level *α*. The conditional type I error rate is computed conditional on the information available at the time of the potential adaptation 8. To understand the distinction between conditional and unconditional type I error rate control, think about a very straightforward situation in which the originally planned sample size of 100 is slightly exceeded simply because there are some patients ‘in the pipeline’ when the trial nears its targeted recruitment. No one would be troubled by this because the standard statistical tests we use are already conditional on the sample size actually achieved. The conditional type I error rate given the actual sample size is *α*. It does not matter that the sample size overrun is an ‘unplanned adaptation’. Contrast this scenario with one in which the sample size is increased after seeing that the observed treatment effect is almost, but not quite, statistically significant at the planned end of the study. The conditional type I error conditional on the observed treatment effect is zero before the adaptation (because we cannot reject the null hypothesis) but larger than zero if the sample size is increased (because we get an additional chance to reject the null hypothesis). Because the conditional type I error rate is increased, so is the unconditional type I error rate. Only when we make an unplanned change before breaking the treatment blind using the ‘lumped’ data from both arms can we validly and sensibly analyze the data.

Adaptations made on the basis of lumped data from both arms is not new. Several authors considered sample size modification using blinded data 9–12. In the context of the analysis of high dimensional data, several authors have considered hierarchical multiple testing procedures, where the order of the hypotheses may depend on the lumped data in a specific way 13–16. In a broader context than clinical trials, 17 proposed looking at lumped data from two groups and selecting the most appropriate rank test to accommodate the observed heaviness of the tails of the distribution. 18 examined the use of a permutation test after using lumped data to select one of a pre-specified collection of models. Our work is closely related to his in the sense that the main tool is a permutation test. We build on 18 by (1) giving necessary and sufficient conditions for a valid test when unplanned adaptations are made, (2) arguing that even when a modification is unplanned, a permutation test can be used, and (3) giving a ‘counterexample’ showing the limitations of the conclusion that is possible depending on what information was used in the adaptation.

## 2 A general strategy

### 2.1.Permutation tests

One useful tool for dealing with unplanned adaptations before breaking the treatment blind is the permutation test. Consider first a nonadaptive setting. A permutation test is conditional on outcome data *Y* = *y*, so the only remaining randomness lies in the treatment assignment vector *Z*. Each possible value *z* for *Z* consistent with the randomization scheme used and the observed numbers of patients in each arm (henceforth called a *consistent z*) is treated as equally likely. We create the permutation distribution by enumerating the test statistic's value *t*(*y*,*z*) for each consistent *z*. We then calculate a *p*-value by referring the value corresponding to the actual assignment vector to this permutation distribution. A permutation test is valid under independence of *Y* and *Z*, namely the conditional distribution of *Y* given *Z* = *z* is the same for every consistent *z*. Loosely speaking, the set of treatment assignments has no effect on the outcome.

Now consider an adaptive setting in which we examine data *X* at the end of the trial but before breaking the treatment blind, and we possibly modify features such as the outcome or test statistic. The data *X* used in deciding whether to make modifications can be very general; it might include data from different end points, covariates, etc. Under the assumption that *X* and *Z* are independent, a permutation test using the modified outcome, test statistic, etc. remains valid. 18 gave a proof of the validity of a permutation test used to select from a pre-defined set of possible models. His proof implicitly assumes countably many potential adaptations 18. Following the technique of 19, we show in the appendix that we control the type I error rate for unplanned adaptations if we control the conditional type I error rate given each possible adaptation (including no modification of the original design). The permutation test does this because any adaptation made before unblinding is a function of the lumped data *X*, and *X* is independent of *Z* by assumption.

Note that the adaptation need not occur at the end of the trial. The design may change on the basis of interim data, and that causes no problem for the permutation test. In fact, making a change early makes adaptations more palatable to regulatory authorities and reviewers because very early changes make it almost as if the trial had been planned that way from the beginning.

There is only one problem with this approach: a permutation test may not have been pre-planned. If it was not, then it is theoretically possible to inflate the type I error as follows. Calculate the conditional type I error rate of the original test given the lumped data; if it exceeds *α*, continue with the original design, whereas if it is less than *α*, change the design and use a permutation test with type I error rate *α*. The problem is that although the conditional type I error rate given the changed design is controlled at level *α*, the conditional type I error rate given *no change* exceeds *α* (see appendix for more details). Nonetheless, the following argument indicates that the degree of type I error inflation is miniscule if the trial is large. If the originally planned analysis was a *t*-test, test of proportions, or other commonly used procedure, the permutation distribution of the *z*-score is approximately normal for large sample sizes. A permutation test is virtually identical to the original parametric test. Because no inflation is possible if a permutation test had been pre-planned, and that permutation test is nearly identical to the original parametric test, we can pretend that the permutation test had been planned from the beginning.

### 2.2.Illustrations using a diet trial

The Dietary Approaches to Stop Hypertension (DASH) trial alluded to earlier was a feeding study randomizing participants with blood pressures in the high normal to stage I hypertension range to three dietary patterns to lower blood pressure 6. In the trial planning stage, DASH investigators debated whether systolic or diastolic blood pressure should be the primary outcome. They settled on diastolic blood pressure, and DASH was not an adaptive trial. But suppose investigators had used lumped data at the end of the trial to make the decision. For instance, they could have looked at the variances of the changes from baseline to end of study in the overall trial, separately for systolic and diastolic blood pressure. Alternatively, they could have looked to see whether there were any apparent outliers for each outcome. As long as the decision was made on the basis of blinded data, the permutation test at the end of the trial on the selected outcome is valid under the strong null hypothesis that there is no effect of diet on blood pressure. Even though they did not originally plan to use a permutation test, they could argue that the originally planned *t*-test is nearly identical to the permutation test for large sample sizes, so it is valid to pretend that the original plan was to use a permutation test.

Now suppose that analysis of the lumped data in DASH revealed outliers that called into question the originally planned *t*-test. They could have changed the analysis to a Wilcoxon test and computed its permutation distribution. Until we break the treatment blind, the conditional type I error rate is no greater than *α* for any permutation test, whether or not the test statistic was changed.

Now suppose that the original outcome for DASH were binary, namely whether a participant's systolic blood pressure increased by 10 mmHg or more. Suppose further that so few participants in the combined trial sample had a 10-mmHg increase that it would be literally impossible for a test of proportions to reach statistical significance. If investigators felt that a 5-mmHg increase was also clinically relevant, they could then change the outcome to the proportion of patients with an increase of 5 mmHg or more. The resulting permutation test comparing a pair of diets with respect to the new outcome is equivalent to Fisher's exact test. Its conditional type I error rate is controlled at level *α* even though the original outcome changed.

In all of the scenarios discussed previously, the adaptation could have occurred at an interim point rather than at the end of the trial. As long as the blind was maintained throughout the trial, the permutation test remains valid. When per-arm sample sizes are known at the time of adaptation, a stratified permutation test is in order, as described in Section 4.

### 2.3.An apparent counterexample and the proper conclusion

It is useful to try to imagine extreme situations that might uncover flaws in the reasoning behind permutation tests to handle unplanned changes. Along that vein, imagine an unscrupulous investigator who examines three end points in a ‘blinded’ manner: (1) mortality; (2) serious AIDS events; and (3) level of experimental drug in the blood. Of course this disingenuous strategy of examining the level of study drug completely unblinds the investigator, who would then be in a position to choose from the other two end points the one that had a stronger observed treatment effect. This would clearly increase the false positive rate for the chosen outcome. The inflation of the type I error rate would be even greater with a larger number of potential end points.

What goes wrong in the counterexample is not the procedure itself but the conclusion. In a trial like this with an unplanned change of end point, the conclusion we would like to make with a statistically significant result is that the treatment improved the adapted outcome. Unfortunately, that is not necessarily the proper conclusion. The assumption underlying the permutation test is that treatment has no effect on the data used in the adaptation. When the result is statistically significant, the proper conclusion is that the treatment has an effect on *at least one* of the outcomes examined. Of course this is a useless conclusion in this counterexample because we know a priori that treatment has an effect on the level of experimental drug in the blood. The fact that the proper conclusion is tempered to the point of uselessness is a good thing because anyone attempting this type of subterfuge pays an appropriate penalty. Still, it is unfortunate that a scrupulous, well-meaning investigator can claim only that treatment affected at least one of the outcomes and not necessarily the adapted outcome. In practice, good judgment is required in the conclusion drawn.

### 2.4.The case of two potential outcomes

It is interesting that the counterexample in Section 2.3 does not inflate the type I error rate when only two potential outcomes are examined. To see that no inflation can occur, note first that the type I error rate is controlled under the strong null hypothesis of no effect of the treatment on the joint distribution of outcomes. Now consider a weaker null hypothesis in which the treatment has an effect on, say, outcome 1 but not outcome 2. For a type I error to occur, the permutation test on outcome 2 must be significant, which has probability *α* or less. Similarly, if the treatment has an effect on outcome 2 but not on outcome 1, the probability of making a type I error is no greater than *α*. Therefore, no inflation of the type I error rate can occur when selecting from two outcomes with no missing data.

One caveat is that if either of the outcomes has missing data, the number of variables examined will always exceed two. That is, we implicitly consider the number of missing observations per outcome in addition to the values of nonmissing observations. When the number of variables examined exceeds two, the potential for inflation of the type I error rate cannot be ruled out. The problem is being caused by the missing data differing by treatment arm being informative about outcome, not really by the fact that an adaptive procedure is used. Even with a nonadaptive trial with a single pre-specified outcome, missing data that differs by arm and is informative about outcome can invalidate a permutation test. Section 3.2 contains more about missing data.

## 3 What data to use for adaptations

### 3.1.Limiting the information examined

The counterexample in Section 2.3 demonstrates that unrestricted blinded data mining results in a useless conclusion. Some of the variables will be informative about the treatment assignments, so the conclusion that the global null hypothesis is false is already known. We must restrict the set of variables and/or analyses under consideration to ameliorate this problem.

One attempt to limit the potential for abuse is to use summary information rather than patient-specific information in the adaptation. After all, the counterexample in Section 2.3 is so problematic because it unblinds individual patients and thereby allows us to choose the end point for which the observed treatment effect or *z*-score is strongest. Knowing only the summary information of the amount of study drug in the blood combined across patients in both arms would tell us little we did not already know. Unfortunately, even summary measures are no safeguard against erroneous decisions. For instance, the sample correlations between the level of the study drug in the blood and the two other end points are summary measures. They do not permit us to determine the assignments of individual patients, but they still allow us to deduce which of the end points has the larger *t*-statistic. Therefore, even though using summary measures is a step in the right direction, we need to think carefully about whether the specific measure selected is informative about the treatment effect.

### 3.2.Amount of missing data or adherence

A seemingly attractive summary measure that might warrant changing outcomes is the total amount of missing data for each outcome. Consider the WAVE trial discussed in the introduction. Recall that there was discussion before the trial about whether percent stenosis or minimum lumen diameter should be the primary outcome. Suppose that for technical reasons, minimum lumen diameter is missing more often than percent stenosis. Whether we should then change the primary outcome to percent stenosis is difficult. Strictly speaking, we can only reject the global null hypothesis that treatment has no effect on the joint distribution of all outcomes considered, including the amount of missing data for minimum lumen diameter and percent stenosis. That would be an unsatisfying conclusion. If clinical trialists were willing to accept that conclusion, they would not object to as-treated analyses. After all, an as-treated analysis is valid under the strong null hypothesis that treatment has no effect on the joint distribution of missingness and outcome. But clinical trialists do not like making such strong assumptions. On the other hand, the problem is being caused by missing data, not by any adaptations. Even if we had only a single possible outcome, we would have problems with interpretation if the amount of missing data were differential across arms. Therefore, with two possible outcomes, it would be hard to argue that changing from the one with a substantial amount of missing data to the one with virtually no missing data would compromise the integrity of the trial. On the other hand, if there were many possible outcomes, one must be wary of the possibility that missing data in one of them differs substantially by arm to the extent that it causes unblinding. That could lead to selecting, among outcomes with very little missing data, the one with the smallest *p*-value.

A related concern arises when considering a sample size increase on the basis of poorer than expected treatment adherence. The problem is that there is no reason to assume that adherence should be the same in the treatment and placebo arms. Rejecting the strong null hypothesis that treatment has an effect on either the outcome of interest or adherence is not helpful. Therefore, making adaptations on the basis of adherence is potentially problematic.

### 3.3.Other potential interpretation problems

There are other scenarios in which seemingly justifiable decisions could make interpretation difficult. For instance, suppose that we plan to use a permutation test (or the signed rank test) on treatment–control paired differences in a crossover trial. We notice that there are a substantial number of people for whom the scores are the same on treatment and placebo, making the paired differences 0. The permutation test or signed rank test excludes such patients, so it might be attractive to increase the sample size to recoup lost power. But this runs the risk of increasing the sample size to detect effects that are not clinically meaningful. For instance, if 80% of the patients have paired differences that are 0, that is compelling evidence that the treatment is not effective in the majority of people. In settings where this implies that the treatment effect is not clinically relevant, the trial's question is probably already answered, and increasing the sample size is not appropriate.

## 4 Incomplete blinding through knowledge of per-arm sample sizes

In the examples we have considered, complete blinding was maintained. In practice, we often know the sample sizes in each arm at interim analyses. The ordinary permutation test is no longer guaranteed to control the conditional type I error rate in this circumstance because the per-arm sample sizes can sometimes give some information about the treatment effect. To see this, consider an admittedly unrealistic example with an immediate binary outcome and only three patients per arm using a one-tailed test at *α* = 0.05. Suppose that after three patients are evaluated, all three have events. If we do not know the number of treatment and control patients, then the difference in proportions might be favorable or unfavorable for treatment. But now suppose we also know that the numbers of patients assigned to treatment and control thus far are 0 and 3, respectively. Then of course we know that all three events are in the control arm. If we continue with the originally planned sample size, the remaining three patients will be assigned to treatment. If none of them has an event, then the Fisher exact one-tailed *p*-value will be 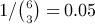, which is statistically significant. If any of them has an event, then the *p*-value will not be significant. Accordingly, the conditional probability of rejection under the original design is Pr (no events among next three patients) = (1 − *p*_*T*_)^3^. If *p*_*T*_ is close to 0, the conditional rejection probability is nearly 1. On the other hand, if we had observed only one event among the first three patients, the conditional rejection probability for the original study would have been 0. In that case, we might have expanded the sample size enough to make the conditional type I error rate arbitrarily close to *α*. It is clear that such a strategy would inflate the type I error rate of the permutation test, which is equivalent to Fisher's exact test.

We can fix the problem of potential alpha inflation of a straight permutation test when sample size information was known by using a stratified permutation test. Instead of considering all possible permutations of treatment labels yielding the given numbers of Ts and Cs at the *end* of the trial, we consider only those permutations yielding the number of Ts and Cs both *before* and *after* the adaptation. In the example of the preceding paragraph, considering only permutations that maintain 0 Ts and 3 Cs at the time of potential adaptation makes the distribution of number of treatment events among those first three patients degenerate at 3. This negates what would otherwise have been a potentially alpha-inflating strategy.

## 5 Discussion

There is an abundant literature on pre-planned adaptive methods but very little on what to do if an unexpected change is made. Regulatory guidance documents are not helpful on this topic because they usually require that adaptive methods be pre-specified. Unfortunately, unexpected things do happen, so it is important to know what the best course is when they do. The only way to control the type I error rate in such circumstances is to control the type I error rate conditional on the information used for the adaptation. If the change is made before unblinding, a very reasonable way to control the conditional type I error rate is to use a permutation test. The permutation test is conditional on even more, namely all of the data; the only randomness is in the allocation of treatment labels. Permutation tests are not uncommon in clinical trials. For binary outcomes, Fisher's exact test is a permutation test. Permutation tests are also nearly identical to parametric *t*-tests when the sample size is large. Therefore, a permutation test is an attractive approach in the face of unplanned changes before breaking the blind.

The methodology is not a panacea. One issue is that its validity requires the strong null hypothesis that treatment has no effect on any of the outcomes used in the adaptation decision. Rejection of this hypothesis does not necessarily mean that treatment benefits the adapted outcome. We gave a sneaky counterexample to show how an unscrupulous investigator could try to unblind by choosing as one outcome the level of study drug in the blood. The joke is on that investigator because the proper conclusion that the treatment affects at least one outcome is useless; we already know that the treatment affects the level of study drug in the blood. Being mindful of possibilities like this, we must try to limit the amount of information used to make any adaptation.

The other down side of our methodology is that although it controls the type I error rate, it cannot eliminate the possibility of bias in the estimate of treatment effect. Without knowing in advance the set of adaptations and the circumstances under which they would be made, there is no way to adjust the treatment effect to eliminate the potential for bias.

Overall, unplanned adaptations will always question the confirmatory nature of a clinical trial, especially if complete blinding is not possible. Unplanned adaptations should be considered only when deemed absolutely necessary. To make the best of a bad situation, remain blinded, limit the amount of data examined and the number of potential adaptations, and choose the best one. Results should never be considered as compelling as if the adapted design had been fixed, but at least the conditional type I error rate will be controlled.

## Appendix Necessity of controlling the conditional type I error rate

We show the necessity, under certain conditions, of controlling the conditional type I error rate if we wish to control the unconditional type I error rate for any unplanned adaptation. Begin with a fixed test function *ϕ*(*Z*) with unconditional type I error rate *α* and whose conditional type I error rate given *X* is not fixed at *α*. For concreteness, let *ϕ* correspond to a *t*-test with small sample size, say six total observations, and let *X* be the lumped data from both arms. Without conditioning on *X*, the type I error rate of the *t*-test is *α*, but its type I error rate given *X* = *x* can vary substantially with *x*. Suppose that our set of potential adaptations is large enough so that after observing *X* = *x*, we can always find another test whose conditional type I error rate given *X* = *x* is arbitrarily close to *α*. For instance, in the *t*-test example, consider the set of all possible sample size increases. It is clear that by choosing the sample size large enough to negate the data seen thus far, we can obtain a conditional error rate arbitrarily close to *α*. Under these conditions, we can find an adaptive rule that inflates the type I error rate, as stated more precisely in the following theorem.

Theorem

Consider a test function *ϕ* whose unconditional type I error rate is *α* and whose conditional error rate given *X* is not almost surely equal to *α*. Suppose that for each *x* we can find a sequence of test functions  such that *E*{*ϕ*_*x*, *k*_(*Z*) | *X* = *x*} → *α* as *k* → ∞. Then we can find an adaptive procedure that inflates the type I error rate.

Proof

Because the unconditional type I error rate of *ϕ*(*Z*) is *α*, *E*{*ϕ*(*Z*)} > *α* for some *X* values with nonzero probability. Begin with test function *ϕ*(*Z*). After observing data *X*, compute E{*ϕ*(*Z*) | *X* = *x*}. If E{*ϕ*(*Z*) | *X* = *x*}*≥ α*, use test function *ϕ*. On the other hand, if E{*ϕ*(*Z*) | *X* = *x*} = *γ* < *α*, switch to test function *ϕ*_*x*,*k*_, where *k* is large enough that E{*ϕ*_*x*,*k*_(*Z*) | *X* = *x*} > *γ*. The type I error rate of the adapted procedure exceeds *α*.

Theorem 1 essentially ensures that a permutation test is the only way to ensure control of the type I error rate for unplanned adaptations if the set of data *X* is unrestricted. In that case, all data other than treatment labels can be used for the adaptation, so the only randomness remaining lies in those labels. However, as mentioned earlier, the permutation test assumes that the treatment has no effect on the joint distribution of *X*, which is totally unrealistic if *X* is completely unrestricted.□
